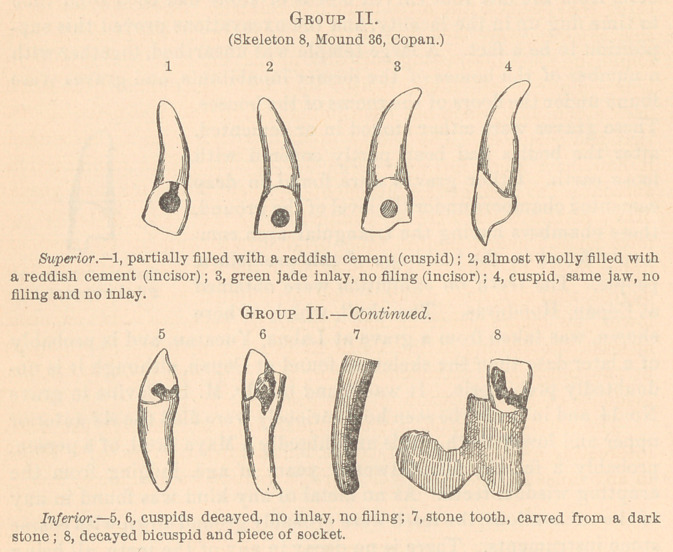# Prehistoric Crania from Central America

**Published:** 1893-12

**Authors:** R. R. Andrews

**Affiliations:** Cambridge, Mass.


					﻿PREHISTORIC CRANIA FROM CENTRAL AMERICA.1
1 A paper read before the American Academy of Dental Science, Boston,
October 4, 1893.
BY R. R. ANDREWS, A.M., D.D.S., CAMBRIDGE, MASS.
At the Peabody Museum, at Cambridge, there is a collection of
archaeological treasures, recently found in Central America, that
have especial interest to the dental profession. These, consisting
of crania, parts of the skeleton, a collection of teeth curiously filed
and inlaid, with a fine collection of pottery and instruments which
were made from bone, stone, and from a volcanic glass, obsidian,
together with carvings and statues, are soon to be on public exhi-
bition. They are being arranged in cases and around the room,
while large numbers of photographs, showing the Bite of the exca-
vations, are arranged within the cabinets upon the walls.
In 1890 the expedition that obtained them was sent to Central
America by Harvard University under the charge of Mr. John Gr.
Owens, a young archaeologist of great promise, who died at his
post of duty after the explorations were about complete. The ex-
pense of this expedition was defrayed by certain wealthy Bos-
tonians. Most of these archaeological treasures were brought from
Copan. Honduras, and certain other ruins found in Yucatan. Mr.
C. P. Bowditch, of Boston, who has been very much interested in
the expedition, has now the charge of the collection. Mr. M. H.
Saville, to whom I am much indebted, and who was with the expe-
dition, states that the site where these things were found was
covered by a growth of old trees, and it was necessary for the
natives to cut these away before the excavations commenced. An
ancient temple of some kind was long supposed to have existed
here, from the fact that carved blocks of stone had been from time
to time dug up in the locality, and the excavations proved this sup-
position to be a fact. A large temple was unearthed, together with
a number of the homes of the former inhabitants, and graves were
found under the floors of the rooms of the houses.
These graves were either stoned in or cemented,
after the bodies had been partly covered with
loose earth. Other graves were found in deep
cemented chambers under the level of the ground,
these chambers having the triangular arch com-
monly found in the buildings of this prehistoric
people. The teeth on exhibition were obtained
at Copan, Honduras. The skull, which is here
shown, was taken from a grave at Labna, Yucatan, and is probably
of a later date than the skeletons found at Copan, although it is un-
doubtedly prehistoric. It was found by Mr. M. H. Saville in grave
No. 14, and in it may be seen how curiously were filed the six anterior
upper and lower teeth. It is undoubtedly a Maya skull, of a person,
probably a female, about twenty years of age, judging from the
erupting wisdom-teeth. As no metal of any kind was found in any
of the excavations, the teeth were probably ground down with coarse
stone instruments. There is no decay in any of the teeth, all being
sound ; but the left superior cuspid is just erupting about a quarter
of an inch inside the arch. It would seem as though these early
people were flesh-eaters, and perhaps cannibals, and that the teeth
may have been filed in this manner for the -purpose of better tear-
ing of flesh. The photograph would imply that the skull was of
considerable capacity, but it recedes very perceptibly from the orbits
upward, so as to appear as though much flattened. The teeth that
were found at Copan, near by, are perhaps more interesting than
the skull. Many of these have small circular pieces of green jade
inlaid in a cavity that has been drilled by a stone or glass instru-
ment in the face of the incisors and cuspids. These inlays are a
little more than an eighth of an inch in diameter, the outer surface
is rounded and brightly polished, and as perfectly fitted as it could
be by the most skilled operator of to-day, with all the modern in-
struments at his command. In a few of the teeth the inlays have
loosened so that it can be taken out, and there appears to be a white
substance, perhaps a cement, between the inlay and the tooth, used
to hold the inlay in place. It would seem that this inlay might be
some mark of distinction, perhaps used in the mouth of a chief or
head man of the people. Some of these teeth are filed and have
no inlay. Some are inlaid and not filed. And some are both filed
and inlaid. Quite a number of the teeth are badly decayed. Much
of this decay appears to be at the cervical border, and in no case
does there appear to be any filling of any kind used to stop decay.
None of them were filled for prophylactic purposes. In the teeth
from skeleton 8, mound 36, found at Copan, two of the teeth that
may have formerly had an inlay were partially filled with some-
thing that seemed like a red cement substance. None of these from
this skeleton were filed, but in the lower jaw of the skeleton was
found the most interesting curiosity in the whole collection to den-
tists,—a lower, left, lateral incisor that has been carved from some
dark stone, and which has been implanted to take the place of one
that bad been lost. The tartar upon it would seem to show that
it had been worn for some time during life. This implantation
antedates Dr. Younger’s experiments by about fifteen hundred years.
Many of the teeth were so completely covered with tartar as to
form masses nearly double their original size, and in one case an
upper molar had the tartar deposited in such a way, and to such a
degree, that it formed a shape that articulated on the gum of the
lower jaw where the teeth had previously been lost. In one case,
at least, the drilling of the tooth to produce a cavity in which to
fit the inlay, had encroached upon the pulp, and there is distinct
evidence of recalcification of pulp tissue at this point.
The whole collection is one of much interest, perhaps the most
interesting evidence of prehistoric dental work that is to be found
in any museum, and it is well worth a visit to Cambridge to see.
				

## Figures and Tables

**Figure f1:**
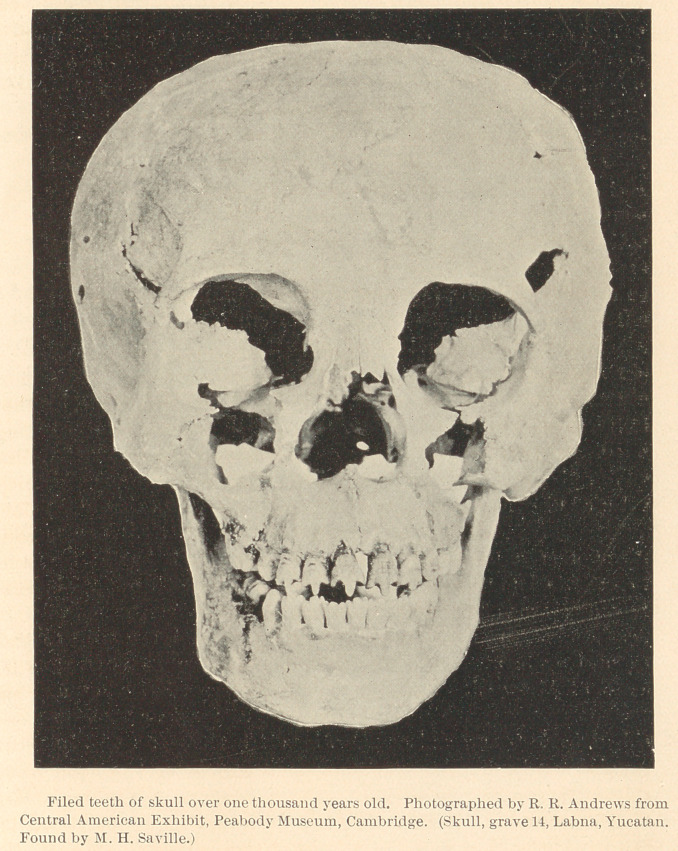


**Figure f2:**
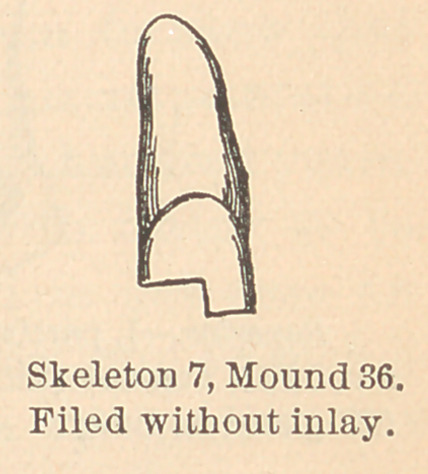


**Group I. f3:**
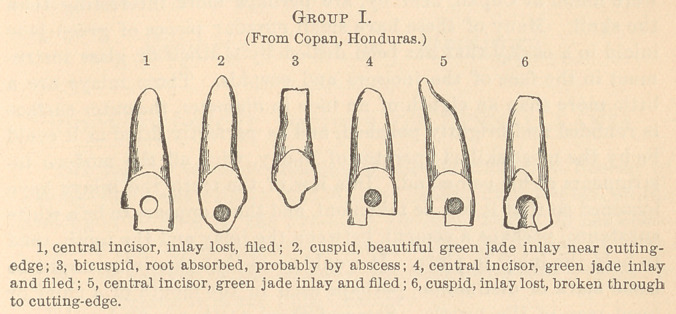


**Group II. f4:**